# Terminal Regions Confer Plasticity to the Tetrameric Assembly of Human HspB2 and HspB3

**DOI:** 10.1016/j.jmb.2018.06.047

**Published:** 2018-09-14

**Authors:** Alice R. Clark, Wilma Vree Egberts, Frances D.L. Kondrat, Gillian R. Hilton, Nicholas J. Ray, Ambrose R. Cole, John A. Carver, Justin L.P. Benesch, Nicholas H. Keep, Wilbert C. Boelens, Christine Slingsby

**Affiliations:** 1 Department of Biological Sciences, Crystallography, Institute of Structural & Molecular Biology, Birkbeck College, Malet Street, London, WC1E 7HX, UK; 2 Radboud University Nijmegen, Institute of Molecules & Materials, Department of Biomol Chem, NL-6500 Nijmegen, Netherlands; 3 Department of Chemistry, Physical & Theoretical Chemistry Laboratory, University of Oxford, South Parks Rd, Oxford, OX1 3QZ, UK; 4 Research School of Chemistry, Australian National University, Acton, ACT, 2601, Australia

**Keywords:** ACD, α-crystallin domain, AP, anti-parallel, MS, mass spectrometry, sHSP, small heat shock protein, TOCSY, total correlated spectroscopy, α-crystallin domain, asymmetric heteromer, heat shock protein, molecular chaperone, polydispersity

## Abstract

Heterogeneity in small heat shock proteins (sHsps) spans multiple spatiotemporal regimes—from fast fluctuations of part of the protein, to conformational variability of tertiary structure, plasticity of the interfaces, and polydispersity of the inter-converting, and co-assembling oligomers. This heterogeneity and dynamic nature of sHsps has significantly hindered their structural characterization. Atomic coordinates are particularly lacking for vertebrate sHsps, where most available structures are of extensively truncated homomers. sHsps play important roles in maintaining protein levels in the cell and therefore in organismal health and disease. HspB2 and HspB3 are vertebrate sHsps that are found co-assembled in neuromuscular cells, and variants thereof are associated with disease. Here, we present the structure of human HspB2/B3, which crystallized as a hetero-tetramer in a 3:1 ratio. In the HspB2/B3 tetramer, the four α-crystallin domains (ACDs) assemble into a flattened tetrahedron which is pierced by two non-intersecting approximate dyads. Assembly is mediated by flexible “nuts and bolts” involving IXI/V motifs from terminal regions filling ACD pockets. Parts of the N-terminal region bind in an unfolded conformation into the anti-parallel shared ACD dimer grooves. Tracts of the terminal regions are not resolved, most likely due to their disorder in the crystal lattice. This first structure of a full-length human sHsp heteromer reveals the heterogeneous interactions of the terminal regions and suggests a plasticity that is important for the cytoprotective functions of sHsps.

## Highlights

• Dynamic behavior of heteromeric sHsps hinders structural biology of cytoprotection.
• Full-length human HspB2/B3 in 3:1 ratio was crystallized and solved at 3.9-Å resolution.
• Assembly is by flexible “nuts and bolts” from terminal regions filling domain pockets.
• N-terminal regions bind in an unfolded conformation into shared dimer grooves.
• IXI/V motifs from unstructured proteins may be sequestered by sHsps during disease.

## Graphical abstract

**Figure:**
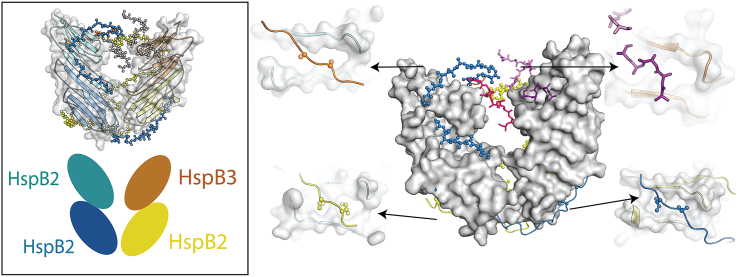

## Introduction

Mammalian small heat shock proteins (sHsps) are among the first responders to cellular stress and therefore play important roles in human health and disease [1], [2], [3], [4]. There are 10 human sHsps (HspB1–10), among which HspB2 and HspB3 have a tissue distribution that supports a protective function in neuromuscular cells: they are components of skeletal and cardiac muscle and are upregulated during differentiation of myoblasts [5], where HspB2 partly co-localizes with HspB3 in the cytoplasm [6], as a “HspB2/B3” complex. HspB3 is present in elongating axons of both motor and sensory neurons, as well as in precursor skeletal muscle [7]. Furthermore, mutations R7S HspB3 and R116P HspB3 [6], [8] are associated with neuromuscular disease. In addition, HspB2/B3 co-localizes with amyloid β deposits in vessels in cerebral amyloid angiopathy [9].

**Fig. 1:**
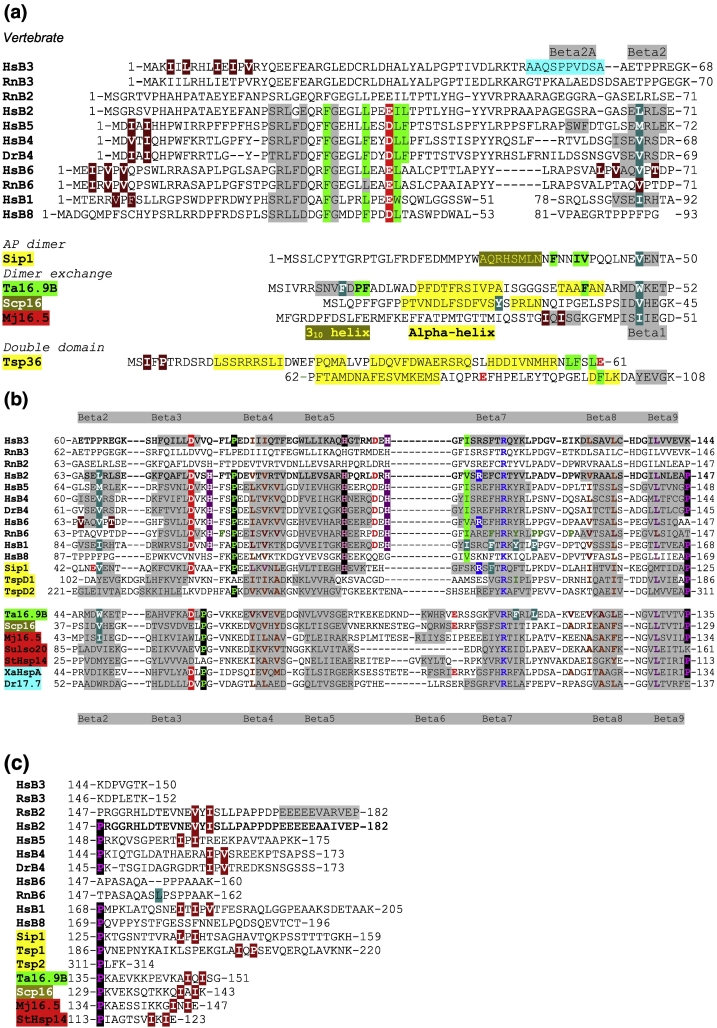
Structure-based sequence alignment of sHsp sequences from vertebrates, worms, plant, fungi, archaea and bacteria annotated with features derived from their X-ray structures (rat HspB2/B3 are included although these have no X-ray structures). The sequence labels and their Uniprot accession codes are as follows: HsB3, Q12988 (HspB3_Human); RnB3, Q9QZ58 (HspB3_Rat); RnB2, O35878 (HspB2_Rat); HsB2, Q16082 (HspB2_Human); HsB5, P02511 (CryAB_Human); HsB4, P02489 (CryAA_Human); DrB4, Q8UUZ6 (CryAA_Danre); HsB6, O14558 (HspB6_Human); RnB6, P97541 (HspB6_Rat); HsB1, P04792 (HspB1_Human); HsB8, Q9UJY1 (HspB8_Human); Sip1, Q20363 (Sip1_Caeel); Ta16.9B, Q41560 (Hs16B_Wheat); Scp16, O14368 (Hsp16_Schpo); Mj16.5, Q57733 (Hsps_Metja); Tsp36, Q7YZT0 (Tsp36_Taesa); Sulso20, Q97W19 (Q97W19_Sulso); StHsp14, Q970D9 (Q970D9_Sulso); XaHspA, Q8PNC2 (Q8PNC2_Xanac); Dr17.7, Q9RTR5 (Q9RTR5_Deira); Salty, Q8ZPY6 (Q8ZPY6_Salty). (A) The N-terminal regions. An alignment of the N-terminal extensions of vertebrate sHsps is shown along with the beta2 strands from the ACDs. The ssNMR structure of HspB5 showed an alternative upstream beta2a strand. The sequences are aligned with respect to the partially exposed core packing beta2 residue **L**-67 in HspB2 and equivalents. The N-terminal **I/V**X**I**/**V** sequences in HspB3, B5, B4 and B6 are indicated. In the structure of N-terminally truncated rat HspB6 (65–162), there is no beta2 strand; instead **V**67 is reaching into β4/β8 pockets of another dimer in the lattice. In the case of N-terminally truncated human HspB6 (57–160), dimers form tetramers by reciprocal exchange of 61-A**L**P**V**AQ**V**P**T**D-70 into partner β4/β8 pockets. In the structure of human full-length HspB6 bound to 14–3–3, the β2-strand is not formed, and N-terminal residues fill βB/β8 pockets. Non-metazoans do not have a shared groove as they have a strand exchange dimer. In the wheat dodecamer, the N-terminal region of one chain of each dimer patches the exposed **W**48 in the beta2 strand and the superfamily-conserved ACD **R**108 using residues **F**10, P12, F13 and F41, respectively. In the yeast Scp16 16-mer, **V**41 packs against **Y**23 in chains B and C. In the archaeal 24-mer, the beta2 strand is patched by the additional beta1 strand (using an **I**X**I** motif). In the tapeworm Tsp36 sequence, the N-terminal patching hydrophobic residues are highlighted in green, and the observed **I**F**P** motif that patches partner β4/β8 pockets is indicated. (b) The ACDs. The alignment of the ACDs comprising Beta2–Beta9 strands from sHsps that have been solved by X-ray crystallography. The residues that line the β4/β8 pockets are shown in brown. Hydrophobic residues that line the shared AP groove in HspB1 are highlighted. When β2 strand is absent, the β3 strand is the edge strand and solvent accessible. In full-length HspB5 (PDB ID 2KLR), the region equivalent to β2 strand is directed away from its own domain allowing a new upstream region (β2a) SWF (Fig. 1a) to H-bond with β3 strand from a partner domain [21]. In a full-length dynamic assembly, it could be envisaged that β3-strand makes alternating interactions with β2 and β2a. The superfamily-conserved **arginine** in β7 is indicated (**R**119 in human HspB2; **R**116 in human HspB3; **R**120 in human HspB5; **R**119 in human HspB6). The AP dimer-conserved **histidine** acts as a pH-sensitive switch. The other highlighted charged residues are generally conserved in most vertebrate sHsp family members, but are not conserved in HspB8. (c) The C-terminal region. The C-terminal regions extend from the ACDs. The sequence highlighted in gray in rat HspB2 is the flexible extension from the assembly structure that is observed in solution by NMR spectroscopy, named the flexible tail or C-terminal extension. The **I**X**I**/**V** motifs are observed by crystallography to be in β4/β8 pockets of a partner ACD. The region between the ACD and the **I**X**I**/**V** motif is ordered in many sHsp structures and includes the hinge region; the sequences are arranged to emphasize the differing lengths of this assembly-critical region. The **I**X**I**/**V** motif is absent in HspB3, B6 and B8. For HspB6, the rat sequence has an insertion SL when compared to the human ortholog. The leucine is bound in the AP dimer-shared groove in a partner dimer in the lattice (Fig. 4d).

sHsps are found in all forms of life and typically are dynamic oligomers that have ATP-independent roles in cellular protein homeostasis, protecting the proteome from uncontrolled aggregation especially during environmental stress [10], [11]. Following recovery, sHsps facilitate the trafficking of non-native proteins to the energy-consuming chaperones and proteolytic machines [3], [12], [13], in some cases *via* the co-chaperone Bag3 [14], [15]. In higher eukaryotes, sHsps have evolved into large families, in which the role of each member in the complex proteostatic networks remains still to be defined [16], [17]. Human sHsps are all cytoplasmic proteins and are particularly prominent in long-lived cells of neuromuscular systems and the eye lens [18], [19]. They are often markers of disease in being components of protein deposits and have a disease phenotype when mutated [1], [20]. Lens crystallins HspB4 (αA-crystallin) and HspB5 (αB-crystallin, which is also found throughout the body), HspB1 (Hsp27) and HspB6 (Hsp20) form a clade of sequences of around 30% identity and share less than 25% sequence identity with other human sHsp family members, which include HspB2 and HspB3 (Fig. 1).

The sequences of sHsps from all kingdoms of life contain a conserved, well-structured “α-crystallin domain” (ACD), flanked by a variable, and generally longer N-terminal region and a shorter more conserved C-terminal region [22], [23], [24]. Crystal structures of rare examples of symmetrical homo-oligomeric sHsps from archaea (PDB ID 1SHS, [25]; PDB ID 3VQK, [26]), bacteria (PDB ID 4ZJD and PDB ID 4ZJA, [27]) yeast (PDB ID 3W1Z, [28]) and a plant (PDB ID 1GME, [29]) show that the ACD β-sandwich, comprising beta strands β2–β9, forms a dimer by β6-strand-exchange: higher assembly is mediated by a sequence motif IXI/V in the C-terminal region (Fig. 1c) of a chain from one dimer filling a β4/β8 pocket in an ACD of another dimer (Fig. 1b) [30]. Some sHsp sequences have the IXI/V motif in the N-terminal region (Fig. 1a); in tapeworm Tsp36 (PDB ID 2BOL) and human HspB6 (PDB ID 5LTW) the N-terminal I-F-P and V-P-V sequences, respectively, fill β4/β8 pockets of partner chains [31], [32] (Suppl. Fig. 1B). The lack of a monodisperse vertebrate sHsp assembly means that atomic coordinates have generally been obtained for smaller assemblies, formed from truncated HspB1, HspB4, HspB5 and HspB6 chains. These structures showed the vertebrate dimer differs in having an anti-parallel interaction (called the AP interface) of elongated “β6 + 7” strands to form an extended beta sheet [10], [33], [34], [35], [36]. The AP dimer has a deep shared groove at the AP interface with sides defined by strands β7 and β2 or β3 [33], [37].

The crystal structure (PDB ID 4YDZ) of a metazoan sHsp, Sip1 from *Caenorhabditis elegans*, shows how 16 AP dimers assemble into a 32-mer by docking C-terminal L-P-I motifs into β4/β8 pockets [38]. The assembly structure shows how the hydrophobic N-terminal sequence 35-FNNIV-39 (Fig. 1a, Suppl. Fig. 1A) from one chain of each AP dimer fills the shared groove [38]. In the crystal structure of the N-terminally phosphorylated human HspB6 dimer complexed with 14–3–3 dimer, there are three hetero-tetramer complexes within the asymmetric unit (PDB ID 5LTW), encompassing three HspB6 dimers formed from chains CD, GH and KL; the N-terminal region of chain G can be traced from 1–38 with residues 27-RLFDQRFG-34 docking into the shared groove of the AP dimer of chains GH [31] (Suppl Fig. 1B). These residues are part of a conserved region of several mammalian sHsps, which includes HspB2 but not HspB3 (Fig. 1a) [30], [39], [40].

Although the crystallized sHsp homo-oligomers are symmetrical, most (particularly mammalian) sHsps are polydisperse. The symmetric sHsps have given indications of flexibility in the system that can contribute to geometric diversity: variation in AP interface register (see Fig. 6 in [41]), the accessibility of the shared groove (see Fig. 7 in [37]), flexibility of the linker between the ACD and C-terminal extension (see Fig. 2B in [28]), patching of β4/β8 pockets by IXI/V motifs from either N- or C-terminal regions and in either direction [42]. Furthermore, in solution these contacts can be heterogeneous and dynamic [43], regulating heteromerization with other sHsps [44]. Similar mammalian sHsp family members are co-expressed in cells, co-assemble, and subunit exchange leading to greater heterogeneity [5], [45]. For HspB1 and HspB6, co-assemblies are built from heterodimers [45], [46]. In the eye lens, HspB4 and HspB5 form a large hetero-oligomer. These many levels of oligomer geometric heterogeneity, fluctuations and interconversions have impeded the atomic study of mammalian sHsps. As the biological function of these sHsps is considered to be derived in part from their irregular assemblies, the challenge in this study was to reduce heterogeneity sufficiently to capture full-length sequences in a crystal lattice while maintaining elements of their signature irregularity and disorder.

Here we have investigated HspB2 and HspB3, which co-assemble into a tetramer with a 3:1 ratio of constituent subunits. This protomer forms the building block for higher oligomer assemblies, consisting of 8–24 chains [47]. This means that although the HspB2/B3 tetramer is polydisperse, it is potentially a more tractable target for structural study than many other sHsps. By assessing the assembly and flexibility of HspB2 and HspB3 from both rat and human, by means of size exclusion chromatography, native mass spectrometry (MS), and NMR spectroscopy, we engineered the human HspB3 sequence to suppress the higher-assembly forms of HspB2/B3. The resulting monodisperse hetero-tetramer was crystallized and solved at a resolution of 3.9 Å, allowing the roles of both the N- and C-terminal regions to be discerned.

## Results

### Making a monodisperse hetero-tetramer of human HspB2/B3

**Fig. 2:**
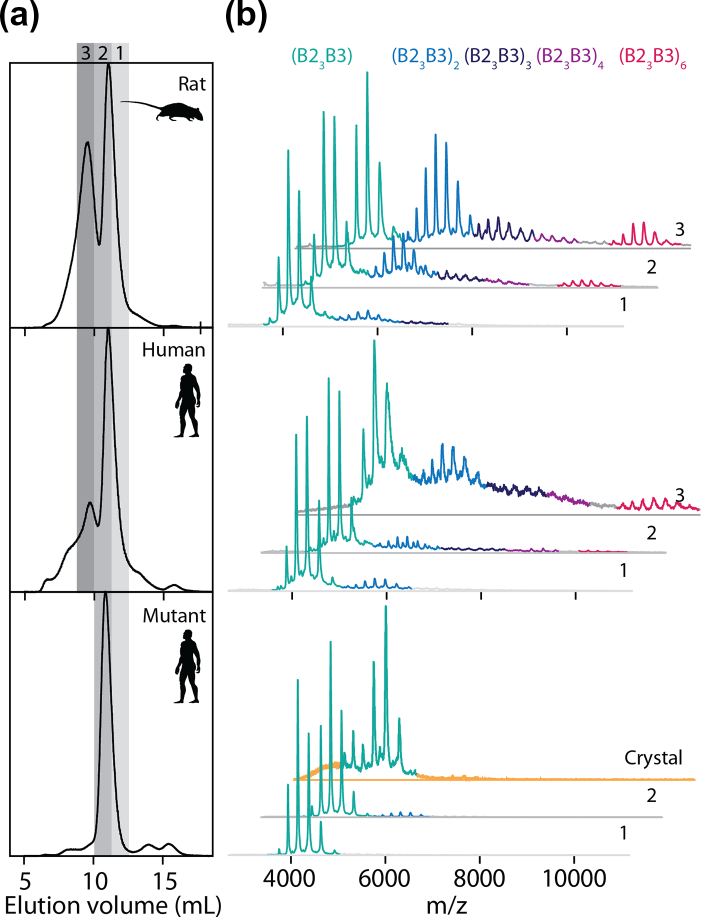
Oligomerization of HspB2/B3. (a) Size exclusion chromatography traces of wild-type rat (upper), wild-type human (middle) and engineered mutant human (lower) HspB2/B3. A dominant peak was observed around 11 mL in each case, with significant amounts of protein eluting at earlier times in the case of the wild-type proteins. Fractions (gray shading, marked 1–3) were collected for further analysis. (b) Native MS data for proteins and fractions as marked in panel A. Multiple charge-state envelopes are observed and can be assigned unambiguously to a protomeric HspB2/B3 tetramer (in a 3/1 ratio of subunits, teal) and multiples thereof (light blue, octamer; dark blue, 12-mer; violet, 16-mer; pink, 24-mer). In line with the SEC data, the wild-type proteins are very heterogeneous. However, the engineered mutant human protein populates almost exclusively the tetrameric state. Native MS analysis of dissolved crystals (orange) demonstrates the retained integrity of the tetrameric state. The raised baseline and shift in charge states is attributable to the effect of residual salt from the dissolved crystals.

**Fig. 3:**
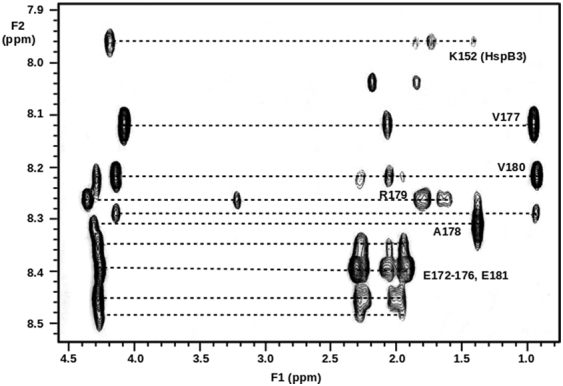
NMR spectroscopy of rat HspB2/B3. NH to α,β,γ-CH region of a ^1^H–^1^H TOCSY NMR spectrum of rat HspB2/B3 with cross-peaks labelled from the flexible and unstructured C-terminal extension of HspB2 and the ultimate C-terminal residue of HspB3 (K152).

We first examined rat HspB2/B3 by means of size exclusion chromatography and native MS. We observed that the protein populated predominately a hetero-tetrameric state, with a 3:1 ratio of subunits (Fig. 2, upper panels). However, higher-order oligomers (8-mer to 24-mers) corresponding to multiple hetero-tetramers were also detected, in agreement with our previous data [47]. This polydispersity is likely responsible for the reluctance of HspB2/B3 to crystallize. Given that C-terminal sequences are known to mediate assembly in other sHsps, and that residues downstream of the IXI/V motifs in HspB1, B4 and B5 are highly flexible [48], [49], [50], [51], whereas the HspB3 sequence terminates shortly after its ACD (Fig. 1c), rat HspB2/B3 was examined by NMR spectroscopy. The solution-phase two-dimensional total correlated spectroscopy (TOCSY) spectrum of rat HspB2/B3 exhibited strong cross-peaks for the C-terminal residues from E172 to P182 of HspB2 (i.e., the last 11 amino acids), indicating that these residues were highly mobile (Fig. 3). Furthermore, the chemical shifts for the α-CH resonances of these residues were very close to the random coil values, indicating that this region was disordered in conformation. The only cross-peak observed for HspB3 arose from the ultimate C-terminal amino acid, K152, indicating that HspB3 does not have a flexible extension, perhaps contributing to the polydispersity of the complex.

We therefore performed size exclusion chromatography and native MS experiments on the human homologous HspB2/3 complex, and the heterologous complexes formed when combining subunits from both species. We found that human HspB2/B3 (Fig. 2, middle panels) and the heterologous complexes (Suppl. Fig. 2) also displayed significant oligomerization into the specific larger assemblies. Although difficult to work with and poorly soluble [47], both human and rat HspB2 in the absence of HspB3 appeared less able to form specific higher-order oligomers (Suppl. Fig. 2). These data, combined with the observation that HspB3 could not be purified alone [47], led us to target HspB3 to engineer a mono-disperse form. To compensate for its lack of C-terminal extension and to enhance its solubility, the highly negatively charged C-terminal extension of human HspB2 171-PEEEEEAAIVEP-182 was added to the C-terminus of human HspB3 (Q12988). This engineered HspB3 mutant co-assembled with HspB2 (Q16082) to form monodisperse tetramers (Fig. 2, lower panel; Suppl. Fig. 3B).

### Structure Solution of the HspB2/B3 hetero-tetramer

Although this mutant version of the human HspB2/B3 hetero-tetramer crystallized, a major problem was obtaining well diffracting crystals. As the majority of crystals that resulted from the initial crystallization hit diffracted to around 8-Å resolution, with none diffracting beyond 7Å, an extensive search for a crystallization condition with improved resolution limits of the data was undertaken. Routine steps included: varying the precipitant concentration and the protein concentration (from 8 to 18 mg/mL), exploring different biological buffer systems and pH, varying the ratio of mother liquor/protein solution and changing the total volume of crystallization drop. When these experiments failed to improve crystal resolution, the search was extended to include *in situ* proteolysis, different cryo-protectants (> 15 tested), different temperatures of crystallization (including nucleation at 21 °C followed by crystal growth 4°C), seeding drops with microcrystals, spiking mother liquor components before setting up drops, including the use of additive compounds into the crystallization conditions (such as detergents, small molecules and ions) and the dehydration of the crystals (with a range of different salts). The best additives were a combination of gamma-polyglutamic acid and GTP. Attempts to find an entirely new crystallization hit, with a more ordered crystal lattice failed. Throughout this screening process, success was measured by the diffraction range of the crystals. More than 100 crystals were tested at three separate synchrotrons over a period of 2 years. The best crystal diffracted to 3.9 Å and the next best crystal diffracted to 4.1-Å resolution. MS confirmed that the crystals preserved the unusual 3:1 HspB2:HspB3 stoichiometry (Fig. 2b).

Solving the structure was challenging due both to the limited resolution of the data and the large size of the asymmetric unit of the crystal comprising three tetramers, giving a total of twelve protein chains. Initially, three chains of one tetramer were identified by molecular replacement with an ensemble of ACDs that comprised Hsp27 (PDB ID 3Q9P), Hsp20 (PDB ID 2WJ5) and HspB5 (PDB ID 2WJ7) overlaid as the search model. The resulting molecular replacement solution contained the AP dimer and one other chain. A bootstrapping method (using model building, phase improvement and rounds of molecular replacement with the improved search model) allowed the identification and modelling of the fourth chain, and finally each of the three tetramers. These are labelled tetramer 1 chains ACDQ, tetramer 2 chains EGFT and tetramer 3 chains IKJV. While the large number of chains was a liability during determination of the asymmetric unit, the 12 chains were then an advantage in refinement, by exploiting the threefold non-crystallographic symmetry in the asymmetric unit throughout refinement and building. The resulting structure was refined using Buster software (Global Phasing) to Rfactor/Rfree of 29.7/31.3, an acceptable outcome for the resolution and the fact that not all the model was identified and built.

Of the 2088 residues expected in the asymmetric unit, 1330 residues were modelled. Side chains for approximately half of these were able to be modelled. The unmodelled peptide, particularly from the N- and C-terminal regions of HspB2 chains D (F,J), is presumably disordered in the crystal, consistent with the resolution being low. Although only 33 residues out of the 85 of the ACDs are identical between HspB2 and HspB3, chains D and Q in tetramer 1 (chains F and T in tetramer 2, chains J and V in tetramer 3) could not be assigned confidently. Therefore, chains D and Q (and their equivalents in tetramers 2 and 3) were modelled as polyAla, along with HspB2/HspB3 conserved prolines. Based on the location in the tetramer, chain Q (T,V) is the most likely candidate for HspB3 and is labelled as such, with chain D (F,J) assigned HspB2 numbering. Details of the equivalent chains in all three tetramers that have been modelled in the asymmetric unit are in Supplementary Table 1.

Segments of N- and C-terminal regions of HspB2 and HspB3 were built *de novo*, into difference electron density that was revealed after refinement using the ACDs only. Some, such as the C-terminal regions of chain A and chain C, have continuous density connecting to the ACDs and a map quality that allowed residue side chains to be modelled. Segments of N-terminal regions were assigned to chains A and C, but the register and direction were uncertain because of the absence of continuous density connecting to the ACD and lack of clear side-chain density in the map. We have modelled as polyAla chain C, residues 20–32 and 36–43 of HspB2, although the numbers and chain direction are uncertain. There are some peptides in the map that could not be assigned to the four chains of each tetramer. These are modelled as chains M, (N,O) in the center of tetramers 1 (2, 3), chains W (Y, X) at the “top” of tetramers 1 (2, 3) and chains 1 (2, 3) (patching β4/β8 pockets of chains Q (T, V) respectively (Supplementary Table 1).

### Description of the HspB2/B3 hetero-tetramer

**Fig. 4:**
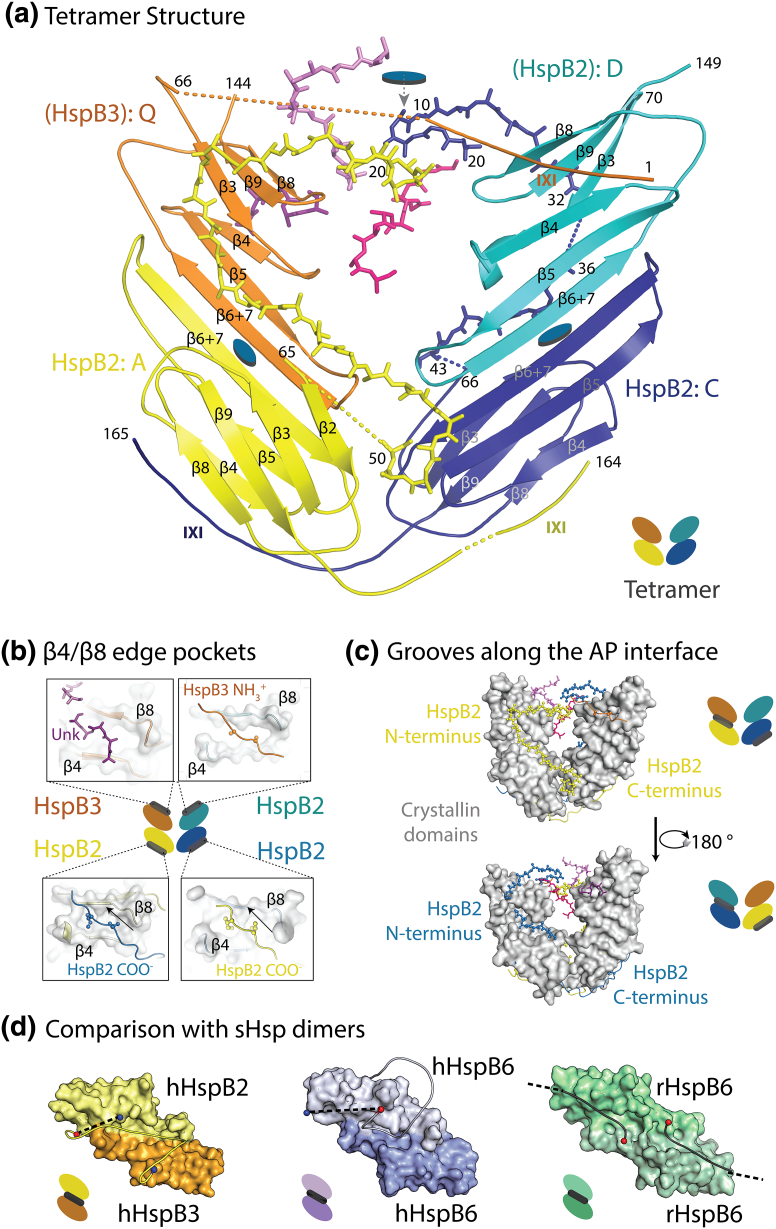
Structure of B2/B3 heterotetramer and comparison with other sHsps. (a) Tetramer 1 of HspB2 and HspB3 (ACDQ) is shown with each chain colored differently showing how the four chains assemble into the tetramer. Chains W (light violet), M (crimson) and 1 (dark violet) are peptide regions external to the ACD but were not able to be assigned to an sHsp polypeptide chain. In ball-and-stick representation, Yellow (chain A) and Blue (chain C) are parts of the HspB2 N-terminal regions. The chains Q and D were not able to be unambiguously assigned and are therefore modelled as polyAla. We propose that HspB3 is most likely the orange chain, with the N-terminal region of this chain reaching across the pseudo-twofold to bind to the pocket of the cyan chain in the adjacent dimer, while chain 1 patches the pocket of chain Q. (b) Pockets. Center is a cartoon of the tetramer, with colors consistent with those in panel A, and the position of the β4/β8 pockets highlighted by thick gray lines. The surrounding panels each shows a zoom into the side view of a β4/β8 pocket, with the extension from a neighbouring chain patching each β4/β8 pocket. The two hydrophobic residues of the IXI motif are shown in ball-and-stick. The peptide direction (N to C) is indicated with the black arrow. (c) The groove along each AP dimer interface is occupied by, in each case, the HspB2 N-terminal region (shown in ball-and-stick representation), from one of the chains within the dimer. The C-terminal regions are shown as cartoon, with the hydrophobic residues of the IXI motif highlighted in ball-and-stick. The cartoon on the right indicates the location of the groove and pocket. (d) Comparison of extensions binding in AP grooves. On the left, a space-filling rendition of the ACDs of HspB2/B3 heterodimer showing how the polypeptide chain (bright yellow thin tube) interacts with the shared AP groove at the dimer interface. The tube of density is interpreted as spanning residue 20 (depicted as a blue ball) to residue 50 (depicted as a red ball) of the N-terminal region of HspB2, with the gap of 16 Å (indicated by the dotted line) between residue 50 and residue 65 (depicted as a blue ball), the first resolved residue of the HspB2 ACD. In the middle, the ACDs of human HspB6 homodimer [31], chain A (pale purple) and chain H (mid purple), showing the resolved N-terminal region (pale blue thin tube) between residues Met 1 and Leu 38, with residues 27–32 of chain G interacting with the shared AP groove at the dimer interface. The distance between residue 38 (depicted as a red ball) and the first resolved residue of the ACD of chain G, Asp 70 (depicted as a blue ball) of 28 Å, is indicated by the dotted line. The first few residues of the N-terminal region that interact with chain G β4/β8 pockets is not visible in this view. Although the N-terminal regions of human HspB2 and HspB6 have some sequence conservation, their resolved conformations differ in part as HspB2 has a β2-strand, whereas the equivalent region in HspB6 is proline rich and folds away from the ACD. On the right, the ACD dimer of rat HspB6 [33] in green showing how in the crystal lattice two C-terminal regions (depicted as dark green thin tubes) from separate ACD dimers interact with the shared groove**.**

Hetero-tetramer 1 comprising chains ACDQ is now described, with chain Q interpreted as HspB3. Each of the four ACDs is a β-sandwich of seven strands β2–β9, as present in other mammalian sHsps. Two HspB2 chains, labelled A and C (Fig. 4a), were identified by their reciprocal exchange of conserved VYI motifs in the region C-terminal to β9 strand (Fig. 1c) docked into partner ACD β4/β8 pockets. This is consistent with solution NMR data showing that the peptide DTEVNE**V**Y**I**SL from the C-terminal region of human HspB2 binds to the β4/β8 pockets of human HspB5 ACD dimer [52]. Chains D and Q are resolved up to and including β9 and form AP mediated dimers with chains C and A, respectively. The symmetry is unusual, reflecting the unique stoichiometry of 3:1 for the HspB2:HspB3 chains. The hetero-tetramer can be described as a dimer of two AP dimers, AQ heterodimer and CD homodimer (Fig. 4a). Reciprocal exchange of the C-terminal VYI motifs into partner β4/β8 pockets of HspB2 chains A and C forms the hetero-tetramer which is bisected by an approximate dyad, that does not intersect the AP dimer dyads (Fig. 4a). The conformation of sequences from the two N-terminal regions of HspB2 chains A and C is similar and related by the hetero-tetramer’s approximate twofold symmetry. A video of ACDQ tetramer colored as in Fig. 4a, in the electron density, is in Supplementary Materials.

Three register shifts have so far been observed at sHsp AP interfaces [43], with register APII being the most common. In the hetero-tetramer structure, APII register is observed at the HspB2 homodimer (chains C and D) and HspB2/B3 heterodimer (chains A and Q) interfaces. Whereas chains A and C form the β2-strand of their respective ACDs, chains D and Q do not, leaving their β3-strand unmasked along the sides of the groove, while increasing the space in the shared grooves. The AP interface has an extended “bottom β-sheet” (β4–β5–β6 + 7-β6 + 7-β5–β4) capped by the two separate ACD (β2)–β3–β9–β8 sheets. The β5/β6 + 7 loops (residues 104–111 in HspB2) bend perpendicular to the sheet similar to that observed in the pH 9 ACD crystal structure of human HspB5 (PDB ID 2WJ7) (see Fig. 4 in [37]). HspB2 C118 is directed below the bottom sheets, which are quite flat in both AP dimer interfaces. In the case of lens α-crystallins (HspB4 and HspB5), these “outer” surfaces of the AP dimer bottom β-sheets are more charged and less hydrophobic than the HspB2/B3 hetero-tetramer: HspB5 has an array of ion pairs, particularly enriched in glutamate and lysine; for example, E99 is bracketed by K92 and K121, whereas these residues are replaced by L95, Q88 and Q117, respectively, in HspB3 (Fig. 1b, Suppl. Fig. 4).

### The assembly interface formed by motif VYI into ACD pockets

There are four sets of β4/β8 ACD pockets in the hetero-tetramer (Fig. 4b). The HspB2 homodimer, formed by reciprocal exchange of C-terminal VYI sequence motifs into partner ACD pockets of chains A and C, is similar to the dimer observed in the B and C chains linking hemispheres together in a yeast sHsp 16-mer (PDB ID 3W1Z) and the homodimer formed from truncated HspB5 chains (PDB ID 3I1G). At the junction of the ACD and the C-terminal region is 149-GG-150, which may favour the reciprocal exchange of VYI by increasing the flexibility of the hinge region. The C-terminal chain crosses from β4 to β8 in the more common direction found in sHsps (also designated as parallel to β8 strand). The HspB3 sequence is C-terminally truncated compared with most sHsp sequences (Fig. 1c), and although it has been engineered here with a longer sequence (from the last 12 highly mobile residues of HspB2), it does not contain an IXI/V motif. However, it does have an extended set of these motifs in the N-terminal region: 3-K**I**I**L**RHL**I**E**IPV**R-15 (Fig. 1a). The map has been interpreted as having 3-K**I**I**L**R7 from HspB3 (chain Q) filling the pockets of chain D (Fig. 4b). It is also feasible that 11-E**IPV**R-15 from HspB3 could be filling this pocket. There is one HspB2 C-terminal region (and hence one VYI motif) per tetramer that is unresolved and potentially unsatisfied. It is too distal from chain Q to patch its β4/β8 pocket. There is density for residues filling the β4/β8 pocket in chain Q, labelled 1–5 (chain 1) of unknown origin (Fig. 4b).

### The N-terminal regions

There are two long tubes of density of similar shape that are interpreted as the N-terminal regions of chains A and C (Fig. 4a and c). The regions most distal from the ACDs each have a hairpin turn numbered as 22–26, which is a sequence-conserved region in sHsps, 22-SRLGE-26 in HspB2 (Fig. 1a). The two tubes of electron density dip into each of the shared grooves of the AQ and CD antiparallel dimers, although they are not connected to any specific ACD (Fig. 4a). To some extent, they are occupying space created by absence of a β2-strand in chains Q and D in each of the dimers. The density is interpreted as the N-terminal region of chain C filling the CD dimer groove, and the N-terminal region of chain A filling the AQ dimer groove (Fig. 4d). Following on from the numbering above, this places residues labelled 34–39 from chain A in the AQ dimer groove, and residues labelled 36–41 of chain C in the CD dimer groove. This interpretation maintains the topology of that observed in the human HspB6/14–3–3 complex (Fig. 4d) and the *C. elegans* Sip1 32-mer [31], [38] (Suppl. Fig. 1). Assuming the strand direction is correct, this is numbered to tentatively indicate the placement of the vertebrate sHsp N-terminal conserved region 34-LPE**E**ILTP-41 of HspB2 into the vicinity of the shared groove (Figs. 1a and 4d). Also in the space between the ACDs of chains Q and D, and the N-terminal regions of chains A and C, are tubes of density built as a 10-residue stretch of polyalanine, labelled chain M and a 12-residue stretch of polyalanine labelled chain W (Fig. 4). It is possible that these chains along with chain 1 are part of the N-terminal regions of HspB3 or HspB2, chain D.

## Discussion

HspB2/B3, the first atomic structure of an sHsp hetero-oligomer, captures many aspects of this protein family’s heterogeneity, a feature that has bedevilled structural biology of vertebrate sHsps. The three-dimensional assembly of four ACDs has aspects of a flattened tetrahedron with the upper and lower flattened surfaces each having a shared groove that binds polypeptide, most likely unstructured parts of the N-terminal regions (Fig. 4). Two of the ACDs (chains D and Q) are more disordered, more exposed to solvent and encapsulate stretches of unfolded polypeptide chain (labelled M, W and 1). The conserved β-sandwich domain supports three types of interface: AP intra-dimer, inter-dimer mediated by the β4/β8 pocket, and the ACD groove, with the N- and C-terminal regions variously employed as partners. The hetero-tetramer is assembled by a combination of motifs (VYI) into pockets (βB/β8), with each AP interface forming a shared groove into which polypeptide chain is docked (Fig. 4). Most protein tetramers have three intersecting twofold axes, an organization that requires precision packing at distinct interfaces and contributes to stability and function. Here the dyads do not intersect resulting in a loosely packed HspB2/B3 hetero-tetramer, for example, in the ACDQ hetero-tetramer, with the ACDs of chains D and Q barely in contact and solvent exposed. Whereas the local intra-tetramer twofold allows the reciprocal exchange of IXI motifs into pockets for chains A and C, a similar interaction between chains D and Q is not possible as they are tilted away from each other. A homo-tetramer of HspB2 would hence likely be unstable due to only two of the IXI motifs being able to reach partner pockets. However, the switching of the IXI/V motif from the C-terminal region to the longer N-terminal region in HspB3 allows a further HspB2 pocket to be filled. A 3:1 ratio would then be the minimal size for a stable oligomer. The unresolved IXI/V motif of chain D could in solution compete for pocket interactions with additional tetramers, to form the higher-order oligomers observed in the wild-type tetramers. Thus, the low symmetry allows weak ACD (AP) interfaces to be supported by sequence extensions filling β4/β8 pockets in non-equivalent ways. The low symmetry also allows a single N-terminal flexible region to insert into a shared groove, an arrangement that leaves the partner N-terminal region available for other interactions. It is possible that the low resolution of the map is due to disorder in the crystals stemming from chains D and Q being randomly occupied by either HspB2 or HspB3. This may reflect the situation in solution and the cytoplasm; whereas there is an assembly strong enough to form a tetramer, it is weak enough to allow free exchange of some partners, in line with the propensity of sHsps to form hetero-oligomers with related family members [45].

A stretch of polypeptide chain interpreted as part of the N-terminal region is situated in each of the shared grooves of the dimers, an interface that is strongly basic and lined with conserved hydrophobic side chains (Figs. 1b and 4). The direction of the chains (A and C) in the AQ (Fig. 4D) and CD dimer grooves, respectively, is the same as the N-terminal extension in AP grooves in *C. elegans* Sip1 32-mer, PDB ID 4YDZ, [38] and the human HspB6/14–3–3 hetero-tetramer complex, PDB ID 5LTW, [31] (Suppl. Fig. 1). Evidence from a previous 3D study on a truncated rat HspB6 chain showed that the AP groove can bind a C-terminal region (Fig. 4d) in the context of a crystal lattice [33]. In full assemblies, it is the N-terminal regions that bind. In the case of the nematode worm, the Sip1 N-terminal sequence 35-**F**NN**IV**-39 (underlined in Fig. 1a) binds to hydrophobic regions in the AP groove [38] (Suppl. Fig. 1A). In human HspB6, the N-terminal sequence 28-LFDQR-32 is bound with the conserved D30 in the vicinity of groove residue **R**115, when the N-terminally phosphorylated S16 is bound into a 14–3–3 cleft [31] (Suppl. Fig. 1B). The proposed interpretation of the new HspB2/B3 heteromer structure places the HspB2 N-terminal region 34-LPE**E**I-38 (underlined in Fig. 1a) in the vicinity of hydrophobic residues in the groove. Bearing in mind that a sterol has been proposed to bind in the groove [53], the AP interface appears to be a tolerant acceptor site, with plastic dimensions due to flexibility of the β2 edge strand. It is possible that this AP groove, lined with arginines and hydrophobic side chains, can serve as a general binding site for substrate proteins, in competition with the N-terminal regions, for example, during chaperone action.

An important functional role for this groove is supported by the location of a superfamily conserved arginine, which in metazoans lies at the borders of the AP dimer groove (Fig. 1b). Mutation of this arginine in several human sHsp family members causes neuromuscular disease [6], [54], [55]. In the human hetero-tetramer, HspB3 **R**116 and the sequence equivalent HspB2 **R**119 lie at the borders of the shared groove. In human HspB3, the **R**116P mutation, which is associated with distal hereditary motor neuropathy, prevents binding to HspB2 when assayed by co-immunoprecipitation (data not shown and [6]). Adjacent to HspB3 **R**116 is Y118, which is mutated to histidine in Charcot–Marie–Tooth disease type 2 [56]. The mutation R7S in HspB3, which is associated with human motor neuropathy [8], is close to N-terminal IXI/V motifs that are proposed to bind a β4/β8 pocket. MS and analytical ultracentrifugation of HspB2/HspB3R7S indicate that the mutation has a minor impact on the hetero-tetramer assembly and stoichiometry (Suppl. Figs. 2 and 3).

Another candidate binding site for destabilized proteins is in the ACD pockets which accept IXI/V motifs in the assembly of all sHsps oligomers. The hetero-tetramer structure has four widely spaced accessible β4/β8 pockets. Solid-state NMR spectroscopy has shown that HspB5 (αB-crystallin) can suppress aggregation of amyloid peptide β1–40 by interactions involving the β4/β8 pockets [57]. The low-activity chaperones HspB2, HspB6 and HspB8 specifically associate with fibrillar amyloid β rather than non-aggregated forms in a hereditary cerebral amyloid angiopathy caused by the rapidly aggregating amyloid β1–40 carrying the “Dutch” mutation 22Glu → Gln [58]. Recent 3D structures of major components of brain amyloid filaments show IXI/V motifs embedded in the ordered parts of the structure [59], [60]. The recombinant amyloid β1-42 dimer fibril 3D structure determined by solid state NMR (PDB ID 5KK3), has a hydrophobic C-terminal region close to the edge of the single fibril, which the authors consider to be a likely feature of secondary nucleation and thus responsible for its neurotoxicity [59]. The region encompasses a reverse IXI/V motif, 39-VVI-41. A 3.4-Å resolution cryo-electron microscopy structure of the common protofilament core of tau filaments purified from an individual with Alzheimer’s disease (PDB ID 5O3L) shows a 306-VQI-308 motif at the start of the first ordered beta sheet [60]. Recent NMR data revealed HspB1 to interact with this region of tau, consistent with binding of substrate IXI/V motifs to sHsp β4/β8 pockets [61]. In such a mechanism of sHsp chaperone action, activity towards these fibril-forming sequences will be modulated by the structural dynamics of both sHsp assembly and the substrate.

The IXI/V motif into pocket interaction is known to be part of the integration of sHsps into the proteostatic cellular machinery. Bag3, which is constitutively expressed in skeletal muscle cells and cardiomyocytes, regulates the cellular pathway that uses the specificity of Hsp70 machinery to recognize and triage misfolded proteins [12], [15], [62]. There is evidence for direct binding of Bag3 to vertebrate HspB8 [14] mediated by the binding of IPV motifs in Bag3 into the β4/β8 pockets of HspB8 [63]. Recently, Bag3 has been shown to use both its IPV motifs to act as a scaffold to physically bring together several sHsps into a ternary complex with Hsp70 which has the ability to refold partially unfolded substrate proteins [64]. HspB2 over-expressed in mammalian cells competes with HspB8 for interaction with Bag3, whereas this binding is negatively regulated by HspB3 [65]. This is consistent with a model in which the binding of the IPV motif in Bag3 is a competition between the β4/β8 grooves of HspB8 and HspB2.

This model of action is consistent with the observation that in the last step in the purification of wild-type human HspB2/B3 hetero-tetramer, a Bag3 column removed all free HspB2, indicating that homo-oligomeric HspB2 has accessible β4/β8 pockets for binding IPV motifs, which are masked when in complex with HspB3. However, in the case of rat HspB2/B3, the complex was less stable as Bag3 also removed HspB2 from the HspB2/B3 hetero-tetramer (data not shown). The greatest difference between the amino acid sequences of rat and human HspB3 is in the N-terminal regions adjacent to the ACD, and in the IPV motif which is TPV in rat (Fig. 1a). These differences may contribute towards weaker binding of rat HspB3 within the hetero-tetramer. In the crystal structure of the ACDQ hetero-tetramer, chain D is numbered as HspB2 with its β4/β8 pocket filled by the N-terminal motif-bearing region of chain Q built as HspB3 (Fig. 4). If this HspB3 N-terminal region patched the pocket of chain D, the hetero-tetramer assembly would be strengthened, whereas intra-chain patching would favour subunit exchange. One possibility is that the “active” component is dissociated HspB2 proffering available β4/β8 pockets, whereas IPV motifs in HspB3 promote hetero-assembly.

The hetero-tetramer structure suggests that it may function in proteostasis by providing accessible binding sites for partially structured and unstructured polypeptide chains whose origins could be destabilized proteins or (intrinsically) disordered regions of other proteins in quality control networks. Unlike the several vertebrate ACD structures currently available, the HspB2/B3 heteromer comprises full-length proteins. By revealing the heterogeneous nature of the interface sites, this structure provides insight into the plasticity of interactions made by sHsps. They reveal the importance of unstructured and flexible N-and C- terminal regions to heterogeneity and probably point to multi-valency of binding substrates, as in the unrelated molecular chaperone SecB [66], [67].

## Materials and Methods

### Preparation of HspB2/B3 + B2tail and HspB3R7S mutant

The cDNAs encoding human HspB2 and HspB3 were cloned into the pET3a expression vector using NdeI–BamHI sites and XhoI–EcoRI sites, respectively. The HspB3 sequence coding for the C-terminal lysine residue was replaced by the sequence coding for the last 12 amino acids of HspB2 171-PEEEEEAAIVEP-182 using the reverse primer - CGGAATTCTCAGGGCTCAACTATGGCTGCCTCCTCCTCTTCCTCTGGAGTCCCAACTGGATCCTTTAC. The R7S mutation in HspB3 was introduced by site-directed mutagenesis using the forward primer GGCAAAAATCATTTTGTCTCACCTCATAGAGATTCC and the reverse primer GGAATCTCTATGAGGTGAGACAAAATGATTTTTGCC.

Protein expression was induced in the *Escherichia coli* BL21(DE3) strain, transformed with pET3a-HspB2/B3 wild type or mutant, by the addition of IPTG to a final concentration of 350 μM and subsequently incubated for 3 h at 37 °C. Cells were lysed by sonication in TEG buffer [25 mM Tris (pH 8.0), 2 mM EDTA, 50 mM glucose] and centrifuged at 16,000*g* for 45 min at 4 °C. The obtained supernatant was fractionated on a DEAE–Sepharose column, using a gradient of 0–1 M NaCl in 25 mM Bis–Tris (pH 6.35) and 2 mM EDTA and, after desalting, further fractionated on a Source 15Q HR 16/10 column using the same salt gradient. Any excess-free HspB2 in an HspB2/B3 batch was removed by briefly incubating the HspB2/B3 batch at room temperature with recombinant GST-Bag3 coupled to Glutathione agarose beads (GE Healthcare, Sweden) in 25 mM Tris (pH 8), 100 mM NaCl and 0.1 mM EDTA, after which the beads were removed. Recombinant GST-Bag3 was obtained with from a Bag3 pGEX4T3 expression construct (kindly provided by H.H. Kampinga, the Netherlands).

### NMR spectroscopy of rat HspB2/B3

NMR spectra of 4.4 mg/mL of rat HspB2/B3 in 90% H_2_O/10% D_2_O, 20 mM phosphate buffer (pH 7.5) and 100 mM NaCl were acquired at 25 ^°^C and a ^1^H frequency of 600 MHz on a Varian Unity Inova 600 NMR spectrometer. The two-dimensional ^1^H–^1^H TOCSY spectrum had a spin lock mixing time of 80 ms and the ^1^H–^1^H nuclear Overhauser effect spectroscopy spectrum had a mixing time of 100 ms.

### Native MS

Proteins were analyzed on a Q-ToF 2 mass spectrometer (Waters Corporation) modified for transmission of high-molecular-mass species [68] using native MS following protocols previously described [69]. Samples were nanoelectrosprayed at concentrations of 10 μM, in 200 mM ammonium acetate (pH 6.9), after retrieval from size exclusion chromatography (Superdex 200 10/300; GE Healthcare) or, in the case of the dissolved crystals, after desalting using 3-kDa MWCO Vivaspin filters (Sartorius). Data were calibrated externally and analyzed using Masslynx software (Waters Corporation).

### Crystallization, crystal parameters, data collection and molecular replacement programs

Crystallization was by hanging drop at 21 ^°^C: 2 μL of protein 10 mg/mL in 25 mM Tris–HCl (pH 6.9), 0.25 mM tris(2-carboxyethyl)phosphine and 2 mM EDTA was mixed with 2 μL of 0.6 M sodium formate, 0.1 M Tris–HCl (pH 7.8), 7.8% gamma-polygutamic acid (Na + form LM) and 2 mM GTP as additives. The best resolution of many data sets collected at a number of synchrotrons was indexed with XDS, and the cell dimensions of the crystal unit cell were determined to be 177.14, 177.14, and 126.46 Å, with angles of 90°, 90°, and 120°, and the space group is *P*3_1_. The dataset was solved using Phaser, revealing 12 chains in the asymmetric unit. X-ray data and refinement statistics are given in Table 1.

**Table 1:** X-ray data and refinement statistics

**Data Collection**	
Space group	*P*3_1_
Cell dimensions	
*a*, *b*, *c* (Å)	177.1, 177.1, 126.5
*α*, *β*, *γ* (°)	90.00, 90.00, 120.00
Resolution (Å)	3.9
*R*_merge_	0.131 (1.163)
*I*/*σI*	6.5 (1.2)
Completeness (%)	99.8 (99.4)
Redundancy	3.7 (3.8)
Wavelength (Å)	0.97949
Mosaicity	0.52
CC 1/2	0.99 (0.36)

**Refinement**	
Resolution (Å)	3.9–78.0 (3.9-4.0)
No. reflections	40,354 (2819)
*R*_work_/*R*_free_	29.7/31.3
Correlation coefficient	0.80
No. atoms	7603
Protein	7603
Ligand/ion	0
Water	0
Wilson *B*-factor (Å^2^)	123.9
r.m.s. deviations (%)	
Bond lengths (Å)	0.01
Bond angles (°)	1.19
Cβ deviations > 0.25 Å	0.08

### Accession Numbers

The coordinates and structure factors have been deposited to the wwPDB with accession number PDB ID 6F2R.

The following are the supplementary data related to this article.

Supplementary Video A video of the ACDQ tetramer, colored as depicted in Fig. 4A, with the electron density map (2fo-fc) depicted in dark blue mesh. Supplementary Video

Supplementary material- Supplementary Table and Figures Image 1
