# Supplementary material for: Terminal Regions Confer Plasticity to the Tetrameric Assembly of Human HspB2 and HspB3
Source: J Mol Biol. 2018 Sep 14;430(18Part B):3297–310. doi: 10.1016/j.jmb.2018.06.047 (PMC6119766; doi:10.1016/j.jmb.2018.06.047)
Supplement: Supplementary file 2 — Supplementary material- Supplementary Table and Figures [file mmc2.pdf]

SUPPLEMENTARY MATERIALS

Supplementary Table 1. Composition of the Asymmetric Unit

**Tetramer 1 chains**	**A HspB2**	**C HspB2**	**D HspB2**	**Q HspB3**	**M/UNK 1-10 Middle of tetramer**	**W/UNK 1-12 Top of tetramer**	**1/UNK 1-5 Patching Q pocket**
**N-terminal region**	Ala20-Ala50*	Ala20-Ala32<br>Ala36-Ala43*		Met1-Ile10			
**ACD**	Ser65-Pro147	Glu66-Pro147	Ser70-Pro147	Glu66-Lys144			
**C-terminal region**	Arg148-Thr155<br>Val157-Leu164	Arg148-Leu165	Arg148-Gly149				
**Tetramer 2 chains**	**E HspB2**	**G HspB2**	**F HspB2**	**T HspB3**	**N/UNK 1-7**	**Y/UNK 1-12**	**2/UNK 1-5 Patching T pocket**
**N-terminal region**	Ala20-Ala50*	Ala20-Ala32<br>Ala36-Ala43*		Met1-Ile10			
**ACD**	Ser65-Pro147	Glu66-Pro147	Ser70-Pro147	Gly67-Lys144			
**C-terminal region**	Arg148-Thr155<br>Val157-Leu164	Arg148-Leu165	Arg148				
**Tetramer 3 chains**	**I HspB2**	**K HspB2**	**J HspB2**	**V HspB3**	**O/UNK 1-6**	**X/UNK 1-12**	**3 /UNK 1-5 Patching V pocket**
**N-terminal region**	Ala20-Ala50*	Ala20-Ala32<br>Ala36-Ala43*		Met1-Ile10			
**ACD**	Ser65-Pro147	Glu66-Pro147	Ser70-Pro147	Gly67-Lys144			
**C-terminal region**	Arg148-Thr155<br>Val157-Leu164	Arg148-Leu165	Arg148				

***Built as PolyAla (numbering very tentative, based on sequence conservation arguments)

Supplementary Figure 1

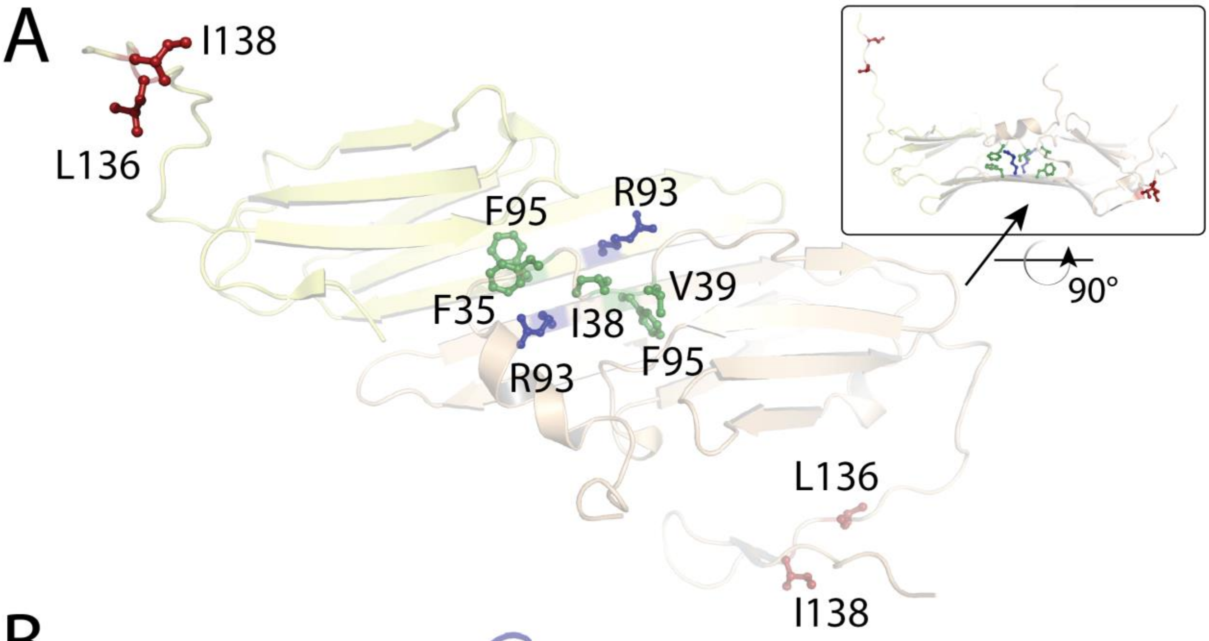

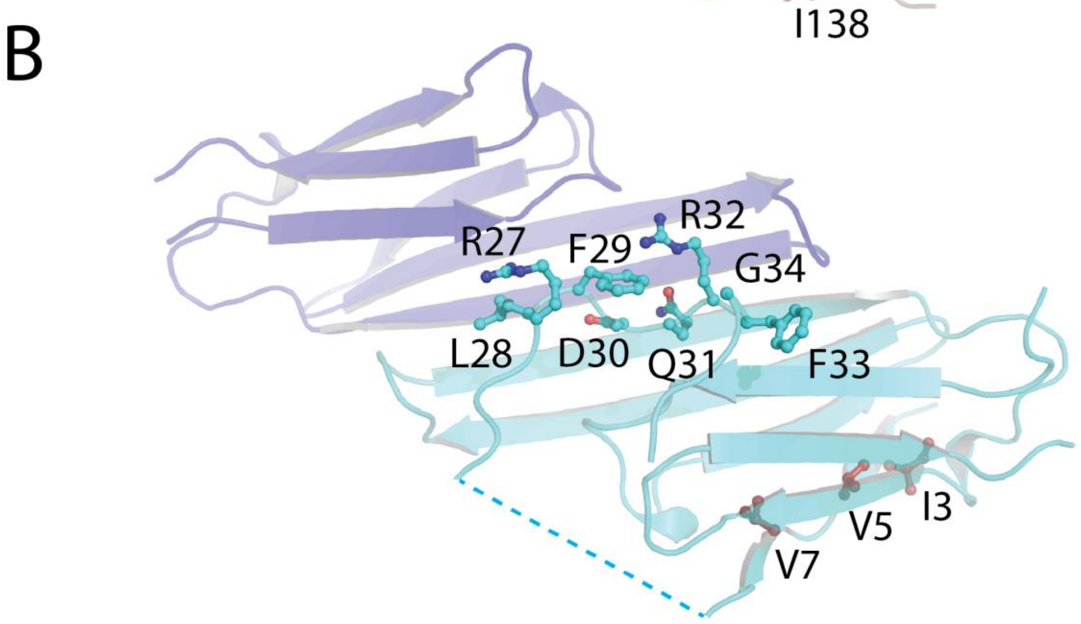

Filling pockets and grooves in assemblies of *C elegans* Sip1 and human HspB6

A) Two views of the AP dimer of chains C (pale pink) and D (pale yellow) of Sip1. The N-terminal residues 35-FNNIV-39 of chain C fill the CD groove, whereby F35:C packs against F95:D, V39:C against F95:C and I38:C squeezes in between R93:C and R93:D. The 136-LPV-138 C-terminal motifs are employed to build the 32-mer.

B) In the AP dimer of human HspB6, the N-terminal residues 27-RLFDQRFG-34 of chain G (light blue) fill the GH shared groove. In HspB6 the IXI motif has swapped from the C-terminal to the N-terminal region. Here residues 3-IPVPV-7 of chain G fill the B4/B8 pocket of chain G.

Supplementary Figure 2

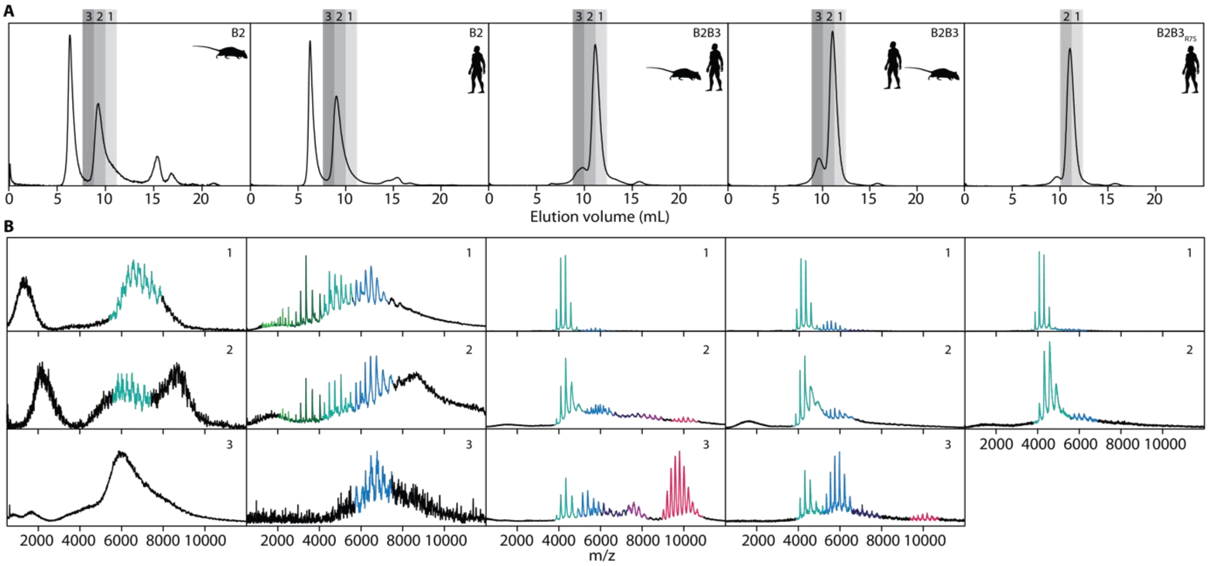

Oligomerisation of HspB2/B3

A) Size Exclusion Chromatography traces, from left to right, of wild-type rat HspB2, wild-type human HspB2, wild-type rat HspB2 – human HspB3, wild-type human HspB2 – rat HspB3, wild-type human HspB2 – R7S human HspB3. Fractions (grey shading, marked 1-3) were collected for further analysis. **B**) Native Mass Spectrometry data for proteins and fractions as marked in **A**. Multiple charge state envelopes are observed and can be assigned unambiguously to: monomer (light green), dimer (dark green), tetramer (teal), octamer (light blue), 12-mer (dark blue), 16-mer (violet), and 24-mer (pink). In the case if the HspB2/B3 combinations, the 3:1 ratio of subunits is preserved.

Supplementary Figure 3

A

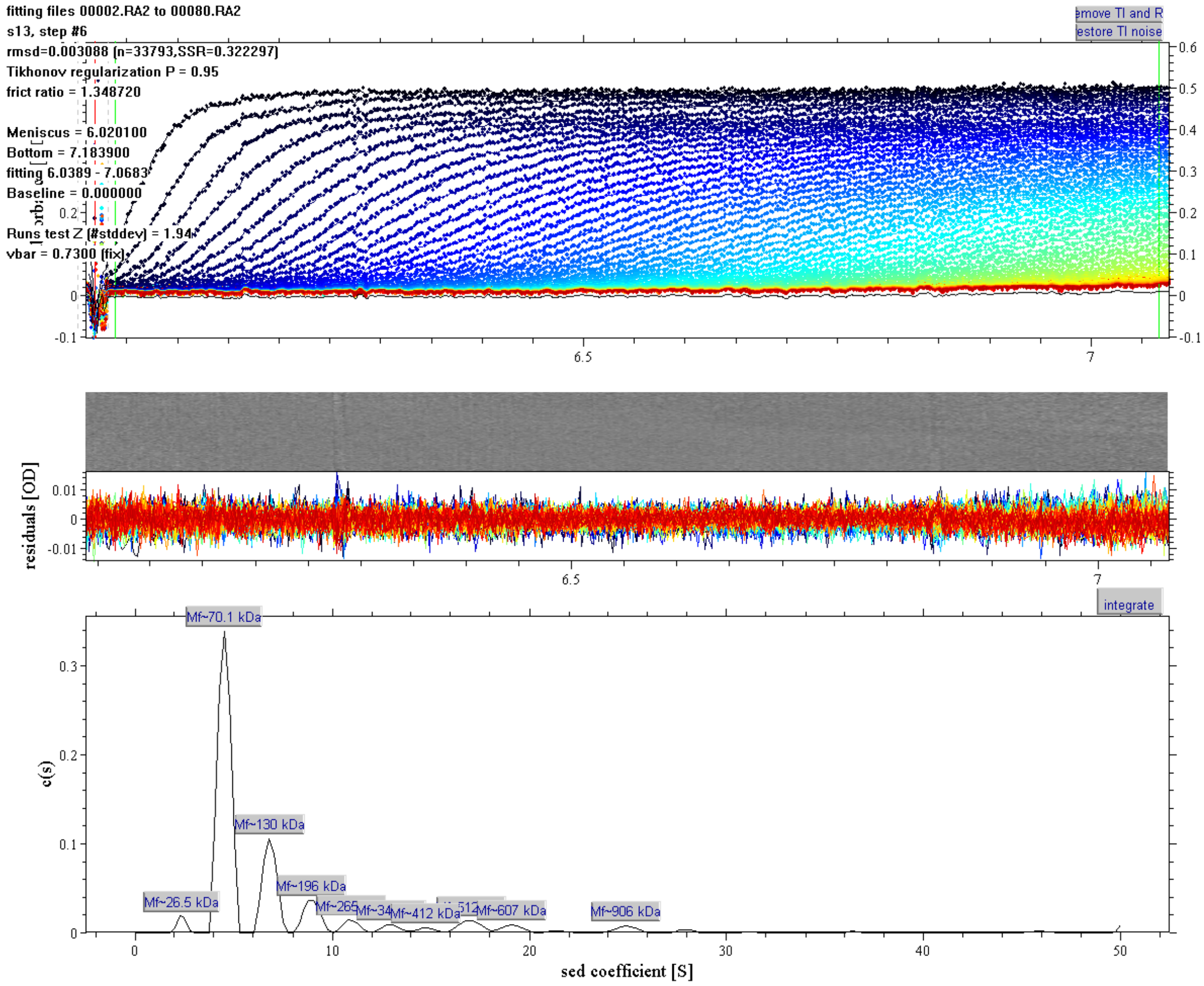

B

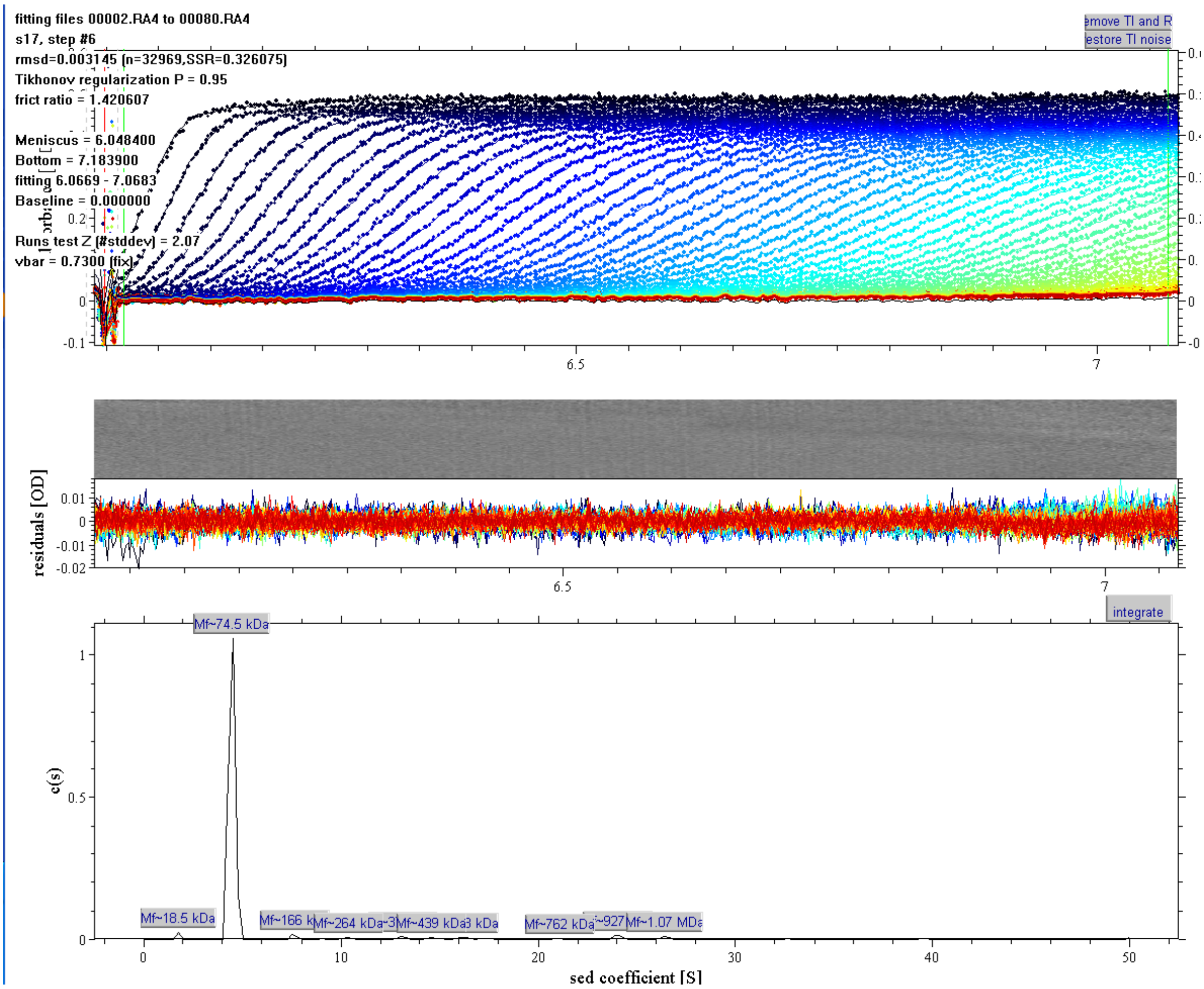
fitting files 00002.RA4 to 00080.RA4
s17, step #6
rmsd=0.003145 (n=32969,SSR=0.326075)
Tikhonov regularization P = 0.95
frict ratio = 1.420607
Meniscus = 6.048400
Bottom = 7.183900
fitting 6.0669 - 7.0683
Baseline = 0.000000
Runs test Z (#stddev) = 2.07
vbar = 0.7300 (fix)
residuals [OD]
integrate
Mf~74.5 kDa
Mf~18.5 kDa
Mf~439 kDa
Mf~762 kDa
Mf~1.07 MDa
c(s)
sed coefficient [S]

C

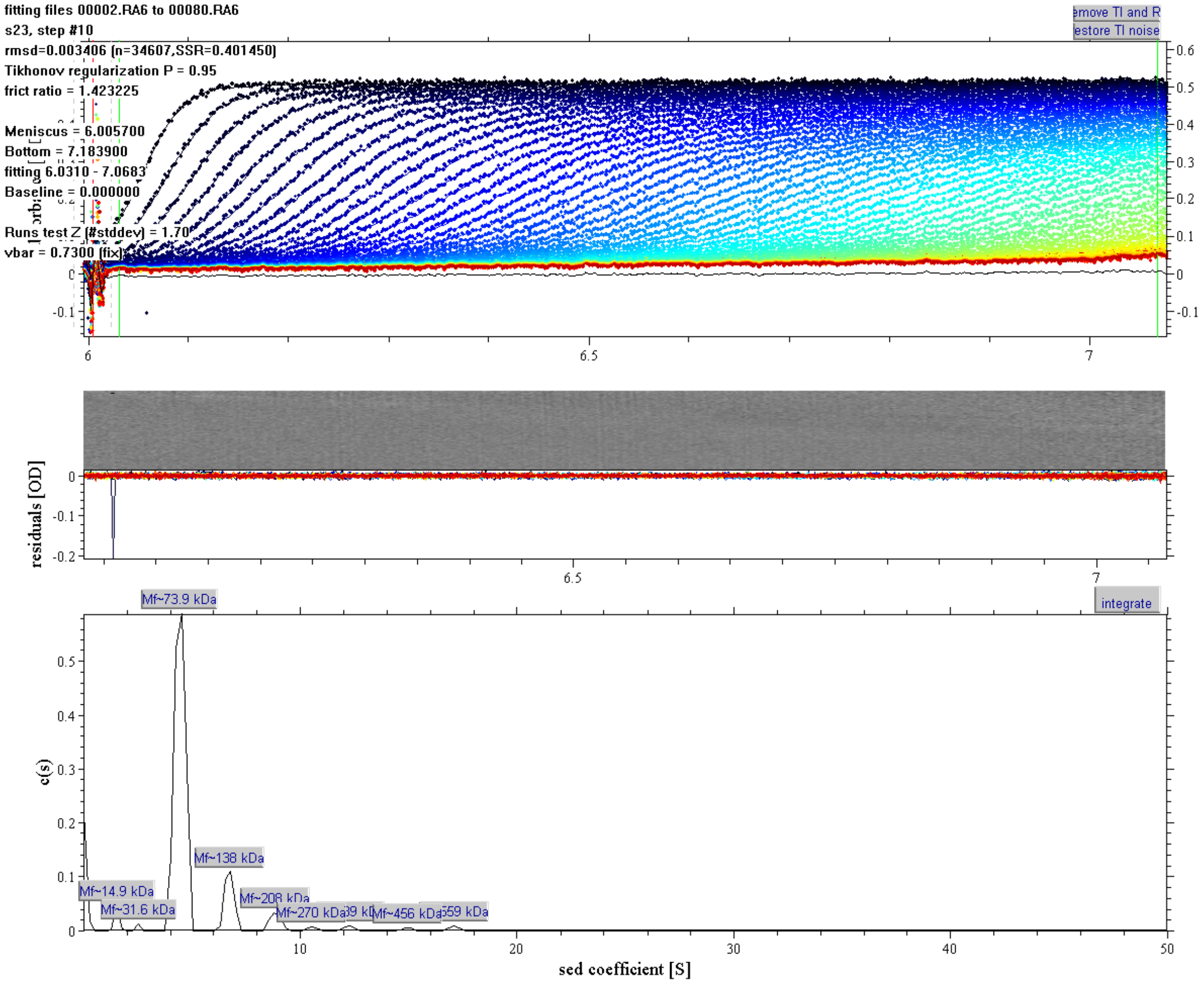

Analytical ultracentrifugation of wild-type and mutant HspB2/B3

A) HspB2/HspB3 A wild type

B) HspB2/HspB3 with engineered C-terminal extension

C) HspB2/R7S HspB3 disease mutant

Supplementary Figure 4

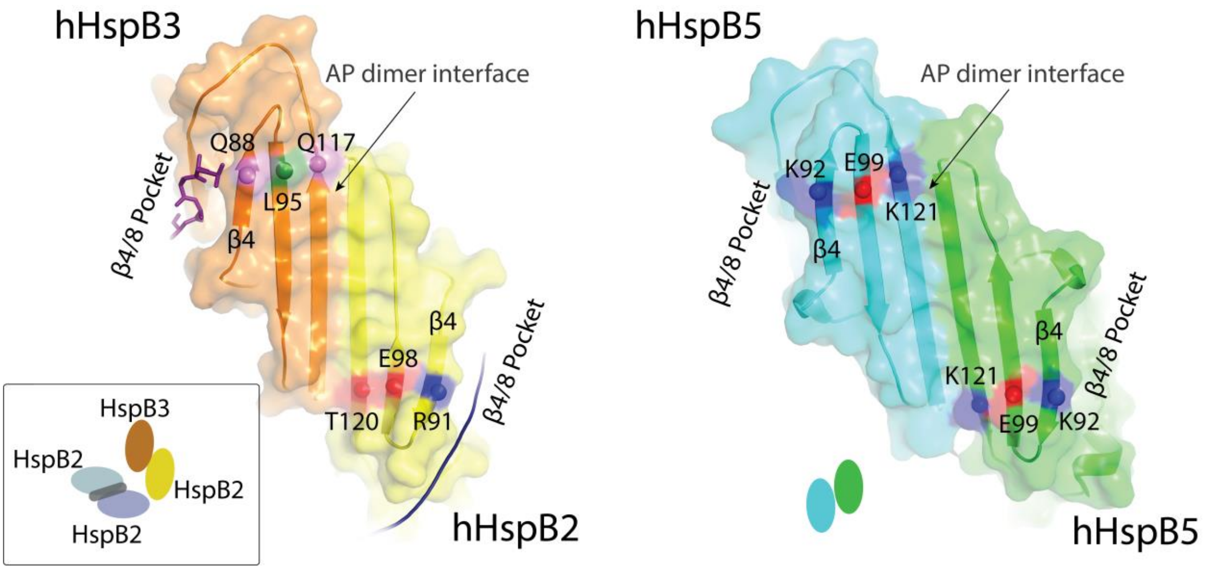

Comparison of the charged residues on the surfaces of the extended β-sheets at the AP interface in human HspB2/B3 and human HspB5 (αB-crystallin). The β-sheet of HspB3/B2 (chains Q and A) is less charged that the corresponding surface of the lens protein human αB-crystallin (HspB5) ACD dimer, **PDB ID 2WJ7.**

Supplementary Video Legend

A video of rotation of the ACDQ tetramer, coloured as depicted in Fig. 4A, with the electron density map (2fo-fc) depicted in dark blue mesh.
